# Web-Based Parent Training Intervention With Telephone Coaching for Disruptive Behavior in 4-Year-Old Children in Real-World Practice: Implementation Study

**DOI:** 10.2196/11446

**Published:** 2019-04-11

**Authors:** Terja Ristkari, Marjo Kurki, Auli Suominen, Sonja Gilbert, Atte Sinokki, Malin Kinnunen, Jukka Huttunen, Patrick McGrath, Andre Sourander

**Affiliations:** 1 Department of Child Psychiatry Turku University Central Hospital University of Turku Turku Finland; 2 Centre for Research in Family Health, Izaak Walton Killam Health Centre Halifax, NS Canada; 3 Strongest Families Institute Halifax, NS Canada; 4 Department of Psychiatry Dalhousie University Halifax, NS Canada

**Keywords:** child mental health, early intervention, parenting education, disruptive behavior, preschool children

## Abstract

**Background:**

Parent training is the most effective approach to the psychosocial treatment of disruptive behavioral problems in childhood. However, no studies exist on how well Web-based training programs work when they make the transition from the research setting to implementation in primary health care.

**Objective:**

The study aimed to examine how the randomized controlled trial (RCT) and implementation study groups of the Strongest Families Smart Website (SFSW) intervention differed in child psychopathology, family demographics and treatment-related factors, such as therapeutic alliance and parents’ satisfaction rates. The intervention was conducted in the pediatric primary health care in Finland.

**Methods:**

The study focused on 232 parents who had taken part in the SFSW intervention, which formed part of a 2-arm RCT study, and 882 families that would participate in the subsequent SFSW implementation study group. Both groups comprised parents whose children displayed high levels of parent-reported disruptive behavioral problems when they were screened in child health clinics at 4 years of age. Parents in both groups were provided with the SFSW intervention, which consisted of a Web-based training program with 11 weekly themes and associated telephone sessions.

**Results:**

Demographic factors or duration of behavioral problems did not differ statistically or clinically between the RCT and implementation groups. Overall, 42.0% (362/862) of children in the implementation group and 35.4% (80/226) in the RCT intervention group had suffered from behavioral difficulties more than 1 year before the screening phase (*χ*_*1*_^2^=3.2; *P*=.07). The mean duration of telephone coaching calls was very similar in the implementation and RCT intervention groups, that is, 38 and 37 min per call, respectively (*t*_279.5_=0.26; *P*=.79). The total time spent on the website of the program was 451 min in the implementation group and 431 min in the RCT intervention group (*t*_318.8_=1.38; *P*=.17). In the RCT intervention group, 52 of the 232 participants (22.4%) discontinued the program before the tenth week, whereas in the implementation group, 109 of the 882 participants (12.4%; odds ratio 2.05, 95% CI 1.4-3.0; *P*<.001) discontinued. Parents in both the implementation (77.1% to 98.5%, 498/742 to 731/742, respectively) and the RCT (64.8% to 98.2%, N=105/162- to 159/162, respectively) groups reported qualitatively similar and high level of posttreatment satisfaction rates in improved parenting skills, expectations, and stress relief. Parents in both groups reported a high level of satisfaction in skills and professionalism of the telephone coaches.

**Conclusions:**

The implementation of population-based screening of Web-based parent training intervention with telephone coaching resulted in good feasibility, fidelity, accessibility, and similar satisfaction level post treatment when compared with intervention in RCT research setting. The discontinuation of treatment in the implementation group was exceptionally low.

## Introduction

### Background

Parent training is the most effective approach to the psychosocial treatment of disruptive behavioral problems in childhood, and there is mounting evidence from randomized controlled trials (RCTs) that such initiatives reduce problems and improve parenting skills [1-3]. It has been proposed that parent training should be provided as primary promotion and prevention in primary care settings [4]. However, only a small proportion of families with these problems benefit from evidence-based treatment programs [5]. The barriers to receiving parent training include the lack of trained staff that can provide interventions; the stigma related to receiving mental health treatment; and the difficulties of accessing and engaging in treatment in terms of costs, time, and location [6,7].

Technology-based parent training programs can offer many benefits over traditional interventions, such as higher fidelity, greater accessibility, convenience, and reduced time and costs [8-11]. Technology-based parent training is not a new innovation because, as early as 1988, Webster-Stratton et al [12] tested videotapes as the primary delivery method for a parent training intervention. Today, technology and internet are integral parts of people’s lives. For example, in 2017, 88% of Finnish people used the internet, including almost every adult under the age of 55 years, and 77% had smartphones, which is one of the easiest ways to access the internet [13]. Accordingly, in the field of parent training as primary care of children’s conduct problems, very recent research has shifted to focus on Web-based training programs. Studies have shown promising efficacy of Web-based interventions in improving child behavior [8,14]. Especially, interactive Web-based programs have been found to be more effective than noninteractive programs [14]. Such Web-based interactive parent training programs could overcome many barriers associated with most traditional programs in the implementation phase, especially concerning the consistency with the original evidence-based intervention [10].

We previously reported the 12-month follow-up study of the first RCT to provide an interactive Web-based parent training program with supplementary weekly phone coaching, the Strongest Families Smart Website (SFSW), using a population-based screening procedure [15]. The 12-month follow-up study showed that the intervention resulted in significant reductions in the level of disruptive behavior problems among 4-year-old children and improved parenting skills. The treatment outcomes remained significant at 24-month follow-up when the intervention group was compared with the control group [16]. However, no research exists about how this model works when the program is implemented in primary health care settings.

Regarding psychosocial interventions, there are 2 different aspects when converting these interventions from the research environment to the *real world*: dissemination and implementation of an innovation in clinical practice. Dissemination refers to how knowledge of the new practices is extended actively and passively, whereas implementation refers to the action of accommodating new practices into real treatment environments [17]. *Implementation gap* refers to the difference between our knowledge of *what* *works* and *how it works* and the application of this knowledge in real-world practice. Research on the implementation of digital mental health interventions into routine care is scarce and studies on such interventions for preschool children are almost nonexistent. A consistent finding is that the vast majority of children with psychiatric problems early in life have unmet needs as only a minority of children with problems are referred and there are substantial delays in contacting specialist services [18]. The great majority of adult psychiatric disorders begin in childhood or adolescence [19]. Therefore, implementation of effective evidence-based treatments in real life plays a key role in child mental health service research [20].

Previous implementation studies have emphasized that certain core implementation components and quality assurance measures ought to be included in the implementation strategy [21]. Typically, the effects seen in the RCT settings decline during implementation [8]. These quality measures are mostly related to the practitioner (referred to as coaches herein) of the program [22,23]. We ensured strict adherence to the same study protocol procedures as in the RCT study by accounting for certain quality measures as explained in the implementation plan (see Methods).

### Objectives

In this study, we implemented our aforementioned RCT study in primary health care settings to see how it would work in the real world. The first aim of this study was to compare certain child and family characteristics of the RCT intervention group with those of the first 882 families who received treatment during the primary care implementation phase. Both groups were based on population-based screening of 4-year-old children with high levels of disruptive behavior [24]. We were particularly interested in finding out whether the level of child psychopathology, duration of problems, and impairment levels were similar when the RCT intervention group and the implementation group were compared. The second aim was to examine the differences between the 2 study groups in certain elements of the program, such as the time the parents spent reading psychoeducational material and completing skill exercises. The third aim was to compare the satisfaction levels in the 2 groups, namely, how the program affected their parenting skills, parental stress, and satisfaction with coaching.

We hypothesized that the screening procedure would also work in the implementation phase and no major differences would be found in the demographic factors or child psychopathology profiles when compared with the RCT study population. We also anticipated no major differences in the content of the program or parental satisfaction levels between the 2 groups.

## Methods

### Context of the Study

The study took place in Finland, a Nordic welfare state that provides its residents with public health services. Finnish child health clinics provide annual checkups that offer universal health care and are attended by 99.6% of children [25]. The clinics try to identify problems that affect families with small children at an early stage and arrange for them to receive appropriate help. All parents are invited to bring their child to the child health clinic checkups about 15 times from birth to the age of 6 years. When the children attend the checkup at 4 years of age, they have reached a suitable stage in their development for identification of disruptive symptoms and provision of early support for families.

### Study Population

**Figure 1:**
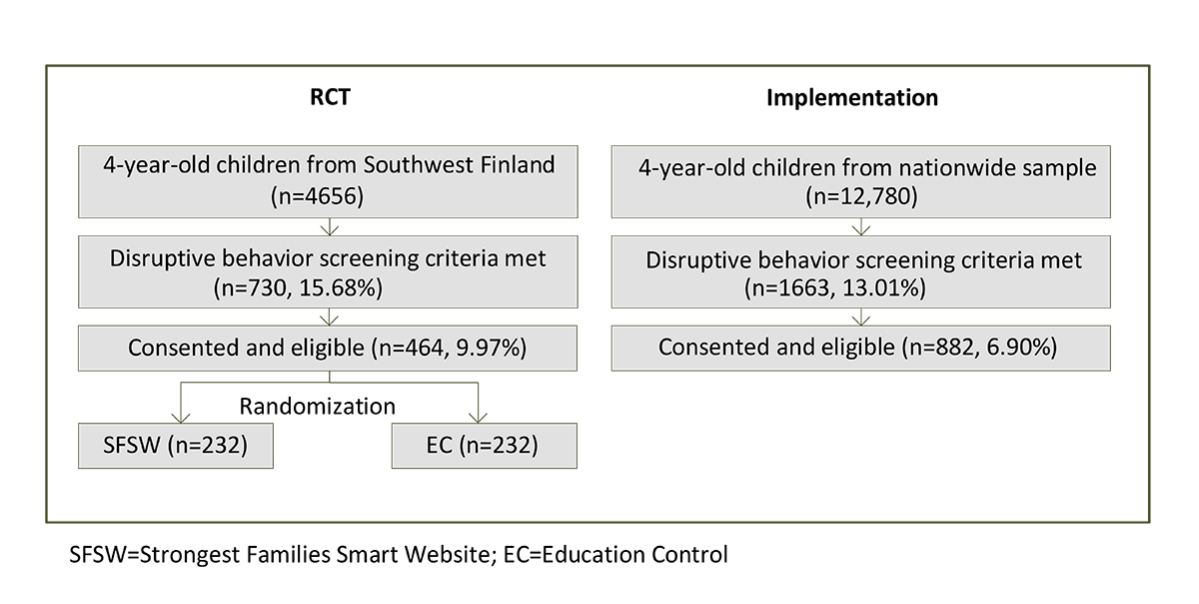
Randomized controlled trial (RCT) intervention and implementation flow charts.

This study compared the intervention group from the RCT study with the first 882 families to receive parent training during the implementation phase. In both groups, all children attending child health clinics checkups at 4 years were screened. Figure 1 shows the flow chart of the study.

The complete RCT study protocol has previously been described in detail [26]. In summary, the study design was an RCT with 2 parallel groups that were stratified by sex with 1:1 individual allocation. From 2011 to 2013, 5 municipalities in Southwest Finland joined the study. After the initial screening of 4656 children, 15.68 % (730/4656) 4-year-old children with high levels of disruptive behavioral problems were identified, and 464 (10.0%) parents were eligible to take part in the study and agreed. These parents were randomized into the SFSW intervention group (N=232) or an education control (N=232). The participants randomized to the intervention received an 11-session, internet-based parenting program that focused on skills for strengthening their parent-child relationships, together with a series of weekly telephone coaching sessions.

The implementation study group comprised the first 882 families to participate in the study following the success of the RCT. Initially, 12,780 children were screened, 1663 (13.01%) children with high levels of disruptive behavioral problems were identified, and 882 (6.9%) of the parents of 4-year-old children were eligible and agreed to take part in the study.

### Implementation Plan

After an RCT study, when converting an evidence-based intervention into practice in a real-world setting, it is essential to tailor discrete multicomponent strategies for the implementation [27]. Here, we identified the core implementation components of our implementation strategy. The following act as implementation drivers [21]:

#### Recruitment, Staff Selection and Training

All coaches were professionals in health care (eg, public health nurses and nurses) and social services (children’s services). In our model, conduction and coaching of the intervention were centralized in the Research Centre for Child Psychiatry at the University of Turku. Each coach was trained on the protocol of the digital program by experienced Strongest Families clinicians. The training consisted of a theoretical background (such as preventive mental health and conduct behavior problems) and rehearsal phone calls. After the basic training, the coaches started the program by recruiting families. Then, they progressed to the closely monitored coaching calls (see below) with the actual families.

Before the utilization of the treatment model (screening and parent training intervention), a half-day training session was organized for the key actors in primary health care (such as public health nurses and doctors) to introduce the background of the program and treatment model and to make announcements and distribute other material.

In primary health care, the child health nurses were kept up to date on the progress of their clients within the Strongest Families program to ensure that they adhered to the parent training intervention model. This included summaries of the screening questionnaires complete with clinical outcome recommendations, updates on families consenting to the program, and a brief summary of the treatment program outcomes. In addition, monthly reports of questionnaire participation rates and numbers of completed treatments and dropouts were sent to child health clinic managers and chief doctors.

#### Ongoing Supervision and Staff Performance Evaluation

We conducted systematic, weekly supervision meetings with the individual coaches and group case conferences, where all coaches reviewed and discussed the families they were coaching. The more experienced coaches acted as supervisors. After each telephone coaching call, the coach assessed his or her own performance on a scale from 4 to 10. If self-assessment was greater than or equal to 6, the supervisor received a message from the digital platform and subsequently discussed the issue with the coach. About 10% of the recorded coaching calls were audited by the coach supervisor and evaluated for competency, with additional training and monitoring of future calls, when indicated.

#### Decision Supporting and Administration

The treatment model was introduced to the stakeholders of children and family services who decided the budget for the region or municipality. All parties signed the jointly funded annual research agreement on the treatment model implementation. Regular contact was maintained with the key actors of the region. The stakeholders received user-friendly monthly progress reports including the number of screened and enrolled families, etc. To integrate the model as a part of primary health care, regular training was conducted and information about the progress of the implementation study was distributed. Moreover, we involved local and national media to increase public awareness of our treatment model and more generally child mental health issues. Centralization of delivery of the digital parent training intervention and the development of the digital platform were the core of the facilitative administration. These strategies strengthened the implementation fidelity, which refers to the degree of adherence to implementation strategy and adaption process into local practices [28].

Of the original municipalities in the RCT study, the City of Turku and Town of Naantali, continued in the program when the implementation study began. Within the first year of the implementation, several municipalities and 2 provinces throughout Finland joined the study. The new treatment model had received publicity in media, and the RCT findings had been presented at national professional and scientific meetings.

The 882 families in the implementation phase came from throughout Finland, specifically the cities of Tampere, Espoo, Kouvola, Kuopio, and Turku; the towns of Lahti, Hyvinkää, Naantali, and Parainen; the municipalities of Kittilä, Mäntsälä, and Tuusula; and 3 provinces, South Karelia Social and Health Care District, Eksote, and Kainuu Social Welfare and Health Care Joint Authority.

A basic study plan was drafted by the research team for the implementation study, and this was introduced to senior child health clinic and health care officials from each of the health care districts. No major revisions were needed to accommodate regional needs.

A significant difference between the RCT and implementation study was that there was no control group, and the first 882 families that met the inclusion criteria were offered the chance to participate in the program. The child health nurses and their supervisors were key to introducing the early intervention program to the families.

### Population-Based Screening Procedure and Recruitment

The screening procedure was similar in the RCT study and implementation phase, and it was integrated into the standard 4-year-old child health checkup visit. First, the supervising child health clinic staff and nurses in the participating areas were informed of the screening and intervention. Then, a 2-hour training session was held in each municipality to introduce the nurses to the background of the program and study plan and what they needed to do. We collected the data for all parents with children turning 4 years after January 2015 from the population register, and letters were mailed to parents in the middle of the month before the child’s fourth birthday. The letters included a short newsletter about the program, the form including questions of demographic information of parents and child, the Strengths and Difficulties Questionnaire (SDQ) [29-31], and instructions for bringing the filled form to the annual checkup at the child health clinic. After the checkup, the nurses mailed the completed form to the research staff, and the answers were entered into the study database and scored with Access software (Microsoft Corp, Redmond, WA, USA). All the questionnaires were then mailed back to the respective child health clinics along with a separate SDQ score sheet, where they were reviewed together with the parents at their next visit to the child health clinic.

The inclusion and exclusion criteria for the implementation study were identical to the RCT trial. Parents were included in the study if their child scored 5 or more on the conduct subscale of the SDQ questionnaire and their answers to the SDQ impact supplement indicated that their child had behavioral problems. Parents were excluded if the child did not live with them because they were under the care of child protection services because of child custody, abuse, or neglect issues; the child did not speak in full sentences; or the parents had participated, or were participating, in other parent training or behavioral treatment. Children were also excluded if they met the diagnostic criteria for autism or a pervasive development disorder, Down syndrome, fetal alcohol syndrome, mental retardation, or a genetic diagnosis that would lead to mental retardation.

If the parents appeared to qualify for the study, they received a phone call from the program’s recruitment staff. They provided a brief introduction to the parent training program and reviewed the exclusion criteria. If the parents wanted to participate, they were directed to the study website to provide their formal consent. A form summarizing the recruitment call was mailed to the referring child health clinic to advise them of the outcome, as the municipalities were obliged to offer alternative treatment options for families not willing to participate in the parent training.

When the parents first logged into the study website, they were presented with the Web-based consent form for the study and a nonbinding program agreement, where the principles for successfully completing the program were outlined. These principles included adhering to the mutually agreed call schedule. The goal of the agreement was to ensure that the parents starting the program were reasonably committed to completing the training, which lasted for 3-4 months.

### Parent Training Intervention

The SFSW intervention consisted of 2 components: the interactive website and the telephone coaching. The parent training program was delivered in a Web-based environment that the parent and coach both had access to, they had scheduled weekly phone calls, and they could also send additional messages via the website. No physical visits or face-to-face communication was conducted at any point during the program.

The program consisted of 11 weekly themes with associated phone coaching, and the program started with an introductory call from the coach after the parent had completed the baseline surveys. This provided an overview of the general objectives of the program and explained the learning methods and need for cooperation. It also set the initial goals for the program and introduced the first of the 11 weekly themes, which was called *notice* *the* *good*. The goals were set in response to the problem behaviors that the child exhibited and that the program aimed to alleviate by teaching the parent how to use positive parenting skills and problem-solving abilities.

After the introduction, the parent was directed to the material for the first Web-based session, completed the associated skill training, and received the next coaching call a week later. The coaches used different verbal techniques during the phone calls, such as discussing different parenting models and role play as well as encouraging parents to practice every day. By using attributional questions, the coach could motivate parents to reflect on their own behavior as well as their child’s. The website tracked the parent’s activities on the site, and if they had not logged on for 2 days, the sites sent them a reminder, suggesting that they log in and complete the activities. The coach was notified if the parents had not been active on the website within 4 days of the last call. They then contacted the parent, encouraged to proceed with the program, and, if necessary, rescheduled the next call. The structured coach interaction with each client and monitoring are the key elements that distinguish coach-guided parent training from other forms of parent training, such as group-based, self-help, or email-assisted programs.

The content of each Web-based session was divided into the introduction, session content, video exercises, troubleshooting, review, and practical application of the new skills. Each section contained interactive and multimedia components, such as exercises and video clips. The parent was encouraged to complete the session by the next phone call. Some sessions also included supplementary material, which was emailed separately. The Web-based sessions followed the chronological order as shown in Table 1.

The sessions were conceptually divided into 3 sections: (1) basic positive parenting skills, (2) practical parenting skills, and (3) reinforcing acquired skills and sustained positive parenting. The aim of the basic positive parenting skills was to refocus the parent’s attention from the child’s problem behavior to noticing the child’s success in everyday life, as reflected in the name of the first session, notice the good. It also aimed to change the parent’s reactions to the child’s behavior from a negative to a positive response. Practical parenting skills focused on applying the basic skills in everyday situations, planning ahead with regard to daily activities, and using the supporting methods to reinforce positive behavior. These included sticker charts, when and then statements, and using time-out as a way to help the child and parent to regulate their emotions.

During the program, the parent learnt to solve problem situations using positive and practical skills and to understand their child better from a developmental and emotional perspective. The final sessions focused on reinforcing how they had applied the skills they had learnt, independent of the coaching support, and how they could sustain those skills beyond the active program.

**Table 1:** Structure of the web-based element of the Strongest Families Smart Website training program.

Session	Key training elements	Parental goals	Supplementary material
Notice the good	Positive and active parenting	Boosts child’s self-esteem; boosts parent’s self-esteem; and changes the parent’s view of their child	—^a^
Spread attention around	Positive, impartial parenting	Strengthens child’s empathy skills	—
Ignore whining and complaining	Positive, self-controlled parenting	Teaches parents self-regulation	—
Prepare for changes	Positive, proactive parenting	Reinforces good daily routines	—
Plan ahead at home	Positive, proactive parenting	Boosts child’s and parent’s self-esteem and involves the child in planning	—
Chart and stickers	Positive, active parenting	Involves the child in planning and reinforces good daily routines	Sticker chart and stickers
Plan ahead outside home	Positive, proactive parenting	Boosts child’s and parent’s self-esteem and involves the child in planning	—
Working with daycare	Positive cooperation and /communication between parent and day care	Helps child to manage and succeed	Daily report card
Time-out	Positive, self-controlled parenting	Teaches self-regulation and consistency	Digital timer
Problem solving, revision, and future application of skills	Positive daily parenting in future	Teaches parents skills to support child development and prepares for upcoming challenges	—
Booster	Skills review	Reminds parents of positive proactive parenting skills	Skills review chart

^a^Not applicable.

### Measurements Used in This Study

Demographic information of family and parents was obtained at the screening phase, and the variables that were included were information about the sex of the child, family structure, and the parents’ birth year and education and whether they had been unemployed. Child variables based on SDQ were collected from the form filled by parents at the screening phase.

Psychopathology was screened using the Finnish version of the SDQ for the parents of children aged 2 to 4 years [30], which is widely used as a screening and research tool and for clinical assessment and outcome evaluation. The SDQ consists of 25 items covering both positive and negative behaviors. These are divided into the 5 subscales of symptoms: emotional problems, conduct problems, hyperactivity or inattention, peer relationship problems, and prosocial behavior. Each subscale consists of 5 questions, and each item is rated on a scale with 3 possible answers: never (0 points), somewhat true (1 point), and certainly true (2 points). The SDQ impact supplement was also used, before inclusion in the program, to determine whether the child had a problem. If the parent said that they felt their child did have a problem, they were asked how chronic the problem was and about the distress, social impairment, and burden that the problem caused to others. The perceived difficulties were assessed with a single question: “Overall, do you think that your child has difficulties in one or more of the following areas: emotions, behavior or being able to get on with other people?” The alternatives were no, minor difficulties, definite difficulties, and severe difficulties.

Each participant’s time on the website was downloaded using appropriate time-out values, including the percentage of primary screens that the participants observed. The duration of therapeutic calls was downloaded and summarized at the end of the treatment. Data on treatment-related factors, program satisfaction, and therapeutic alliance questions were collected and provided to the research team at the end of treatment. The treatment-related factors covered 3 domains. The first was where the coaching took place (at home, at work, or in another place) and how often the coaching calls were received (every week/almost every week, a couple of times/once during the program, or never). The second was where the materials were read (at home, at work, or in another place), how often they were read (daily/almost daily, a couple of times a week/once a week, or never), and whom they read the material with (alone or with the child’s other parent). The third was the parents’ satisfaction with the program and therapeutic alliance questionnaire that explored their relationship with the program coach. These used a 5-point Likert scale (strongly disagree, disagree, not agree or disagree, agree, and strongly agree) and contained 13 propositions and 3 sections, covering the family program ( the program matched my expectations, I would recommend the program to my friends, if they were in need of similar help, in case I would need help in future, I would enroll to the program again, generally speaking, how content have you been with the Strongest Family program?), the effect of the program on their parenting skills ( I have learned skills, which have been helpful to me as a parent, I trust more my abilities to act as a parent, my relationship with my child has improved, my stress levels have been relieved), and the family coach ( the coach respected my views on parenting, the coach was professional, the coach encouraged problem solving, I could form a successful working relationship with the coach).

### Ethical Approval

Ethical approval was received from the research ethics boards of the Hospital District of Southwest Finland and our Canadian program partners, the IWK Health Centre, Halifax, Nova Scotia, for the RCT study. We received ethical approval from the University of Turku for the implementation study.

### Data Analysis

To explore the differences between the implementation and the RCT intervention, we used the Pearson Chi-square test for categorical variables or the Fisher exact test, if the assumption of expected counts was violated. The assumption of equal variances and residuals normality was tested for continuous variables. If the assumptions were valid, the independent group *t* test was used. If normality assumptions failed, the *t* test was repeated after logarithmic transformation. If the log-transformed variable was not reaching assumptions, the Mann-Whitney U test with normal approximation for large samples (Z test) was applied. The effect sizes were estimated by the Cramér V for Chi-square test and Fisher exact test and by the Cohen *d* for *t* test and Z test.

The odds ratios (ORs) with 95% CI were estimated using logistic regression to examine the associations between discontinuation in the implementation and RCT groups. The statistical significance was classified by a 2-way *P* value of <.05. The statistical analyses were conducted using SAS statistical software version 9.4 (SAS Institute Inc, Cary, NC, USA).

## Results

The RCT group (N=464 including both intervention and control group) was screened from a population of 4656 children who were 4 years old (9.97%), whereas the implementation group including 882 children was screened from 12,780 children who were 4 years old (6.90%; *χ*_*1*_^2^=45.0; *P*<.001). Of those 232 who were in the RCT intervention group, 52 (22.4%) discontinued the program before the tenth week, whereas the respective figure for those 882 in the implementation group was 109 (12.4%). The logistic regression indicated that parents in the implementation group were twice as likely to complete the program than in the RCT intervention group (OR 2.05, 95% CI 1.4-3.0; *P*<.001).

### Family Demographics and Child Psychopathology

Tables 2 and 3 shows the family demographic and child psychiatric information for the 882 children (553/882, 62.7% boys) in the implementation group and the 232 children (142/882, 61.2% boys) in the RCT intervention group. No differences were observed in family structure, parental age, or education level between the 2 groups. When the SDQ psychopathology of the children was compared, the groups had very similar scores. However, the implementation group had more moderate or severe difficulties (53.1%, 467/880; *P*=.02; effect size=−.071) and more severe impairment based on SDQ impact score (mean 1.1, SD 1.4; *P*=.006; effect size=0.214) than the RCT intervention group (44.4%, 103/232; mean 0.8, SD 1.2. It was notable that 42.0% (362/862) of the children in the implementation group and 35.4% (80/226) of the children in the RCT intervention had suffered from behavioral difficulties for more than a year before they were assessed.

### Treatment-Related Factors

As shown in Tables 4 and 5, most of the treatment variables were very similar in both groups. The telephone coaching calls were mostly received at home. Parents who took part in the RCT intervention phase were more likely to receive calls and read the material at work than the parents in the implementation group. Overall, the implementation group received an average of 11 calls (SD 2.2), whereas the RCT group received an average of 10 calls (SD 3.3; Z=3.41; *P*<.001) over the course of the program. The mean duration of the calls was very similar in both groups, lasting 37 to 38 min per call. The time spent on the website during the whole 11-week program was a mean of 451 (SD 174) min in the implementation group and 431 (SD 207) min in the RCT group (*t*_318.8_=1.83; *P*=.17).

**Table 2:** Family and child factors in the implementation (N=882) and randomized controlled trial intervention (N=232) groups. Statistically significant values (P<.05) are shown in italics.

Family factors			Implementation, n (%)	Randomized controlled trial intervention, n (%)	Chi-square test (df)	*P* value	Effect size^a^
**Family and parent variables**							
	**Sex of the child**				**0.2 (1)**	**.68**	**0.013**
		Male	553 (62.7)	142 (61.2)			
		Female	329 (37.3)	90 (38.8)			
	**Family structure**				**0.3 (1)**	**.58**	**−.017**
		Two biological parents	720 (81.9)	192 (83.5)			
		Other	159 (18.1)	38 (16.5)			
	**Maternal age at childbirth**				**0.4 (1)**	**.55**	**0.018**
		Up to 24 years	112 (12.8)	26 (11.3)			
		25 plus years	764 (87.2)	204 (88.7)			
	**Paternal age at childbirth**				**2.4 (1)**	**.12**	**0.048**
		Up to 24 years	69 (8.2)	11 (5.1)			
		25 plus years	778 (91.9)	207 (95.0)			
	**Maternal education**				**1.0 (1)**	**.31**	**−.031**
		Comprehensive school or lower or secondary education	339 (38.9)	98 (42.6)			
		College/university degree	532 (61.1)	132 (57.4)			
	**Paternal education**				**0.1 (1)**	**.71**	**0.012**
		Comprehensive school or lower or secondary education	445 (54.7)	115 (53.2)			
		College/university degree	369 (45.3)	101 (46.8)			
**Child variables based on Strengths and Difficulties Questionnaire**							
	**Difficulties**				**5.5 (1)**	***.02***	**−.071**
		Minor difficulties	413 (46.9)	129 (55.6)			
		Moderate or severe difficulties	467 (53.1)	103 (44.4)			
	**Length of difficulties**				**3.2 (1)**	**.07**	− **.055**
		Less than 1 year	500 (58.0)	146 (64.6)			
		1 year or more	362 (42.0)	80 (35.4)			

^a^The effect size is measured by Cramér V.

**Table 3:** Family and child factors in the implementation (N=882) and randomized controlled trial intervention (N=232) groups. Statistically significant values (P<.05) are shown in italics.

Family factors	Implementation, mean (SD)	Randomized controlled trial intervention, mean (SD)	Z-test	*t* test (df)	*P* value	Effect size^a^
Total score	15.5 (4.6)	14.9 (4.4)	—	1.76 (1108)	.08^a^	0.139^b^
Emotional score	1.9 (1.8)	1.7 (1.5)	−1.1	—	.29^b^	0.128
Peer problems score	2.4 (1.7)	2.3 (1.6)	—	1.16 (1109)	.25^a^	0.081
Prosocial behavior	5.9 (1.9)	6.0 (1.8)	—	−0.80 (1112)	.43	−.059
Hyperactive score	4.9 (2.5)	4.7 (2.3)	—	1.14 (1111)	.26	0.086
Conduct score	6.2 (1.3)	6.2 (1.3)	—	0.49 (1112)	.62^a^	0.042
Impact score	1.1 (1.4)	0.8 (1.2)	−2.8	—	*.006^b^*	0.214

^a^The *P* value after logarithmic transformation by *t* test.

^b^The *P* value was determined by the Mann-Whitney U test with normal approximation for large samples.

**Table 4:** Treatment-related categorical factors in the implementation (N=882) and randomized controlled trial intervention (N=232) groups, excluding 21 parents who did not have any coaching calls. Statistically significant values (*P*<.05) are shown in italics.

Treatment-related factors					Implementation, n (%)	Randomized controlled trial intervention, n (%)	Chi-square test (df)	Fisher exact test	*P* value	Effect size^a^
**Where calls were received?**										
	**At home**						**2.6 (2)**	**—^b^**	**.27**	**.055**
			Every week/almost every week		563 (76.2)	125 (82.2)				
			Couple times/once during the program		148 (20.0)	23 (15.1)				
			Never		28 (3.8)	4 (2.6)				
	**At work**						**5.8 (2)**	**—**	**.054**	**.082**
			Every week/almost every week		118 (16.0)	31 (23.3)				
			Couple times/once during the program		146 (19.8)	30 (22.6)				
			Never		475 (64.3)	72 (54.1)				
	**Other place than home or work**						**3.1 (2)**	**—**	**.21**	**.60**
			Every week/almost every week		54 (7.3)	7 (5.7)				
			Couple times/once during the program		306 (41.4)	42 (34.4)				
			Never		379 (51.3)	73 (59.8)				
**Where materials were read?**										
	**At home**						**0.5 (2)**	**—**	**.79**	**.023**
			Daily/almost daily		50 (6.8)	11 (7.0)				
			Couple times a week/once a week		680 (92.0)	144 (91.1)				
			Never		9 (1.2)	3 (1.9)				
	**At work**						**—**	**0.0001**	***.002***	**.117**
			Daily/almost daily		2 (0.3)	1 (0.7)				
			Couple times a week/once a week		193 (26.1)	57 (39.9)				
			Never		544 (73.6)	85 (59.4)				
	**Other place than home or work**						**4.2 (2)**	**—**	**.12**	**.070**
			Daily/almost daily		7 (1.0)	1 (0.8)				
			Couple times a week/once a week		152 (20.6)	16 (12.8)				
			Never		580 (78.5)	108 (86.4)				
**Whom were materials read with?**						
	**Alone**						**0.8 (2)**	**—**	**.77**	**.024**
				Daily/almost daily	41 (5.6)	10 (6.4)				
				Couple times a week/once a week	661 (89.5)	141 (89.8)				
				Never	37 (5.0)	6 (3.8)				
	**With the other parent of the child**						**0.7 (2)**	**—**	**.69**	**.029**
				Daily/almost daily	10 (1.4)	2 (1.4)				
				Couple times a week/once a week	423 (57.2)	78 (53.4)				
				Never	306 (41.4)	66 (45.2)				
**Completed part of the program**							**24.3 (3)**	**—**	***<.001***	**.148**
	Only introduction part				16 (1.8)	14 (6.0)				
	Basic skills (weeks 1-4)				51 (5.8)	18 (7.8)				
	Functional skills (weeks 5-9)				45 (5.1)	24 (10.3)				
	Revision and future application of skills (weeks 10-11)				770 (87.3)	176 (75.9)				

^a^The effect size is measured by Cramér V.

^b^Not applicable.

**Table 5:** Treatment-related continuous factors in implementation (N=882) and randomized controlled trial intervention (N=232) groups, excluding 21 parents who did not have any coaching calls. Statistically significant values (*P*<.05) are shown in italics.

Treatment-related factors	Implementation		Randomized controlled trial intervention		Z-test	*t* test (df)	*P* value^a^	Effect size^b^
	Mean (SD)	Range	Mean (SD)	Range				
Number of calls	11 (2.2)	2-14	10 (3.3)	1-14	3.41	—^c^	*<.001*	.146
Duration of call (min)	38 (10.3)	3-102	37 (13.5)	2-82	—	0.26 (279.5)	.79	.021
Duration of sign-ins (by staying on the website, min)	451 (174)	72-1330	431 (207)	29-1362	—	1.38 (318.8)	.17	.107

^a^The *P* value was determined by Mann-Whitney U test with normal approximation for large samples.

^b^The effect size is measured by Cohen *d*.

^c^Not applicable.

### Parents’ Satisfaction

As Table 6 shows, the participants in both groups reported high levels of satisfaction with how the program had improved their parental skills. The satisfaction questionnaire was filled by 744 of 882 participants (84.4%) in the implementation and 162 of 232 participants (69.8%) in the RCT intervention phase. In the implementation group, 83.4% (619/742) to 98.5% (731/742) reported high satisfaction with various aspects of the program, and in the RCT group, 82.7% (134/162) to 98.2% (159/162) were very highly satisfied. When they were asked about the overall impact of the program, 88.4% (658/744) of the parents in the implementation groups said it had matched their expectations and 94.0% (699/744) said it had matched their needs. The respective figures for the RCT intervention group were 82.7% (134/162) and 93.8% (152/162). In addition, 77.1% (498/742) of implementation group parents and 64.8% (105/162) of the RCT parents reported that the program had reduced their stress. Finally, both groups reported very high levels of satisfaction (96.8% [719/743] and 100% [162/162], respectively) with the skills and professionalism of the telephone coaches and their relationships with them. We conducted a sensitivity analysis on the satisfaction-related factors, where we excluded 2 municipalities from the implementation sample that participated in the RCT study (altogether 159 cases). The results were not qualitatively different from the tests on the complete data.

**Table 6:** Satisfaction-related factors in implementation (N=882) and randomized controlled trial intervention (N=232) groups, excluding 21 parents who did not have any coaching calls. Statistically significant values (*P*<.05) are shown in italics.

Satisfaction-related factors^a^			Implementation, n (%)	Randomized controlled trial intervention, n (%)				Chi-square test (df)	Fisher exact test	*P* value	Effect size^b^
**Overall impact: family program**											
	**The program matched my expectations**							**3.96 (1)**	**—^c^**	***.047***	**−.066**
		Disagree/neutral	86 (11.6)	28 (17.3)							
		Agree	658 (88.4)	134 (82.7)							
	**The program met my needs**							**0.004 (1)**	**—**	**.95**	**−.002**
		Disagree/neutral	45 (6.1)	10 (6.2)							
		Agree	699 (94.0)	152 (93.8)							
	**I would recommend the program to my friends, if they were in need of similar help**							**1.01 (1)**	**—**	**.31**	**.033**
		Disagree/neutral	25 (3.4)	3 (1.9)							
		Agree	719 (96.6)	159 (98.2)							
	**In case I would need help in the future, I would enroll to the program again**							**0.16 (1)**	**—**	**.69**	**−.013**
		Disagree/neutral	97 (13.0)		23 (14.2)						
		Agree	647 (87.0)		139 (85.8)						
	**Generally speaking, how content have you been with the Strongest Family program?**							**—**	**0.20**	**.18**	**.020**
		Disagree/neutral	20 (2.7)		3 (1.9)						
		Agree	724 (97.3)		159 (98.2)						
**Overall impact: effects on parenting skills**											
	**I have learned skills, which have been helpful to me as a parent**							**—**	**0.09**	**.18**	**−.047**
		Disagree/neutral	11 (1.5)		5 (3.1)						
		Agree	731 (98.5)		157 (96.9)						
	**I trust more in my abilities to act as a parent**							**0.39 (1)**	**—**	**.53**	**.021**
		Disagree/neutral	86 (11.6)		16 (9.9)						
		Agree	656 (88.4)		146 (90.1)						
	**My relationship with my child has improved**							**0.03 (1)**	**—**	**.87**	**.006**
		Disagree/neutral	123 (16.6)		26 (16.1)						
		Agree	619 (83.4)		136 (84.0)						
	**My stress levels have been relieved**							**0.31 (1)**	**—**	**.57**	**−.019**
		Disagree/neutral	244 (32.9)		57 (35.2)						
		Agree	498 (77.1)		105 (64.8)						
**Direct impact: family coach**											
	**The coach respected my views on parenting**							**—**	**0.04**	**.09**	**.063**
		Disagree/neutral	16 (2.2)		0 (0)						
		Agree	726 (97.8)		162 (100.0)						
	**The coach was professional**							**—**	**0.16**	**.40**	**.034**
		Disagree/neutral	19 (2.6)		2 (1.2)						
		Agree	723 (97.4)		160 (98.8)						
	**The coach encouraged problem solving**							**—**	**0.19**	**.55**	**.028**
		Disagree/neutral	17 (2.3)		2 (1.2)						
		Agree	725 (97.7)		160 (98.8)						
	**I could form a successful working relationship with the coach**							**—**	**0.04**	**.07**	**.067**
		Disagree/neutral	24 (1.2)		1 (0.6)						
		Agree	719 (96.8)		161 (99.4)						

^a^Disagree/neutral combines strongly disagree, disagree, and not agree or disagree. Agree combines agree and strongly agree.

^b^The effect sizes are measured by Cramér V.

^c^Not applicable.

## Discussion

### Principal Findings

This study describes the content and process of implementing a Web-based parent training program with telephone coaching in comparison with the RCT intervention. The program is unique because it is based on screening children from the general population during routine child health clinic checkups at the age of 4 years. First, the findings show that the characteristics of families recruited to the RCT study and the implementation groups were very similar. Second, the duration and content of the Web-based training and phone coaching were similar in both groups. Third, the satisfaction rates did not differ between the groups. Finally, against expectations, the discontinuation rate of the program was higher in the RCT group than in the implementation group.

The screening procedures that were used to identify children at risk during both the RCT and implementation phases resulted in similar profiles for families and child psychopathology. The only exceptions to the profiles were that the implementation group had 2-fold more moderate or severe difficulties (53,1%, 467/880) and more severe impairment based on SDQ impact score than the RCT intervention group (44.4%, 103/232). This result may indicate that the parents of the children with more severe disruptive behavior are more motivated to seek help and to keep up with the program than those of the children with minor behavioral problems. The same outcome has been found previously [24]. We suggest that the parental motivation related to the volume of the problem may act as one of the implementation drivers.

The study documented the duration and content of the 2 key elements of the remote parent training program, which were Web-based training and phone coaching. Both sets of parents spent about 80 min engaged in the program each week, when the figures for website use and coaching were combined. However, this did not include the time parents spent practicing the positive training skills with the child during the program, which was the key goal of the Web-based content and phone coaching.

The study showed high and similar parent satisfaction rates in both groups. The level of satisfaction with the program, how the program affected parenting skills, and how the parents worked with the phone coaches remained very high when the program made the transition from the RCT to the implementation phase. Most of the parents felt they had been able to form a successful working relationship with the coach.

Finally, the study shows that less than 12,7 (112/882) of parents discontinued the program before the tenth week in the implementation group, which was almost 2-fold lower (24,1%, 56/232) than that in the RCT study. This indicates that the Web-based parent training programs supplemented with telephone coaching may result in high success. In our study, we identified core implementation drivers to facilitate the implementation process. The intervention fidelity was ensured in the implementation phase, by centralization of the conduction and coaching of the intervention, systematic quality assurance enabled via a digital platform, and supervision. Moreover, both ongoing training of the primary health care staff and stakeholders and facilitative administration, that is, regular meetings, user-friendly reports, and involving media, were likely to lead to successful implementation.

To our knowledge, the SFSW intervention is the first Web-based parent training program to use population-based screening to select its subjects and provide telephone coaching. Other digital interventions have reported using automated messages or emails to interact with parents [8,12]. During the program, the coach and the parent formed a working relationship, which was crucial for meeting mutually agreed goals [32,33]. Telephone coaching has a number of benefits: it enables real-time problem solving with the parent, provides direct feedback on how the parents adopt strategies and skills, and motivates the parent to continue the learning process. In addition, the website tracking and telephone coaching worked together to ensure sufficient exercises were conducted by the parent between the weekly sessions.

The findings of this study are important in a number of ways. They can help to inform best practice in implementing interventions that target large numbers of parents in a wide geographical area. They can also help to tackle the problems that are inherent with traditional parent training group interventions, such as drop-out rates and practical and resource issues. We have demonstrated that it is possible to replicate the success of an RCT at the implementation stage if the families are motivated and the structure of the program provides the right elements and meets their needs. Our experience also underlines that the ongoing supervision of those delivering the coaching element of the training is essential. It is notable that the use and adherence rates of digital mental health interventions might be lower in real-world settings, as implemented outside of research settings [34]. Our findings show that discontinuation of the intervention in the implementation group was exceptionally low and users were highly satisfied with the intervention.

Web-based interventions provide various opportunities for extending services to people with mental health [9] and behavioral problems, as they provide an effective way of reaching people, including the parents of children, as with our program. Moving interventions outside traditional clinics and into people’s daily environments, like the internet, can facilitate better access to mental health services [14]. Web-based interventions can also remove the barriers associated with the face-to-face interventions and enable people to seek help for mental health problems without fear of being stigmatized [7,35,36]. From the families’ point of view, digitally delivered programs can offer faster and more flexible services without the need for transport, juggling work schedules, arranging childcare, or the practical cost of accessing services. On the basis of the evidence, Web-based telephone-assisted interventions for children with disruptive behavioral problems could play a significant role in parent training by engaging families into low-threshold primary care services to promote the mental well-being of families and children [10,14,15].

As nearly all Finnish families use the child health clinic services, our treatment model reaches a very high proportion of the population. This enables early access to the low-threshold intervention, which is in accordance with the core principles of the Finnish child health clinic system, that is, early prevention, early detection, and increasing the awareness of physical and mental health. The elements of the treatment model constitute a new form of preventive intervention for disruptive behavior.

### Limitations

There were some limitations to our study. We did not have any information about changes in the children’s psychiatric problems or changes in parenting skills in the implementation group, which would have told us if the real-life implementation was as effective as the RCT study setting. We only had information about the time they spent on the website and telephone coaching, their satisfaction with the program, and how well they felt it met their needs. It must be noted that neither the therapeutic alliance nor the parent satisfaction questionnaire was validated for the Finnish settings. The alliance questionnaire was based on the original WAI (Working Alliance Inventory), whereas the satisfaction questionnaire was self-composed. Both the Web-based element and the coaching calls encouraged the parents to practice the skills they learnt in real life, but we do not have information about how much the parents actually did this. Information from public health nurses would have helped us to evaluate how the intervention was integrated into the primary health context. Finally, it is important to note that this study is an implementation study and not a sustainability study. However, the implementation sample in this study has been gathered up to 3 years after the RCT study ended. Sustaining an intervention in practice over time is different from scaling up interventions in practice. To maintain the effects observed in this study over a longer period may require additional strategies.

### Conclusions

This study has important implications for planning low-cost, low-threshold, early, population-based, and evidence-based interventions in future. Our study revealed very similar findings between the RCT and implementation groups with regard to the profiles of the families and child psychopathology, the duration and content of the Web-based training and phone coaching, and the parents’ satisfaction rates. The discontinuation rate was lower and disruptive behavior was more severe in the implementation group. These findings may suggest that the parents of the children with more severe disruptive behavior are highly motivated to receive help and complete the program. In this sense, parental motivation may be seen as an implementation driver. To our understanding, the successful implementation in this study constituted of numerous factors and their seamless interaction: the convenient and easy-to-use Web-based format with supplementary telephone coaching by trained professionals, sufficient training for stakeholders and primary health staff on the procedures, the psychosocial evaluation, and the support that the program families would receive. Moreover, we suppose that the central role is played by the primary health care, which in Finnish child health clinics has a potential to reach almost 100% of the children. We believe that this program provides a number of advantages over traditional parent training models, which face enormous challenges such as practical, resource, and retention issues, and that it could provide a blueprint for other cost-effective preventive and early intervention child mental health programs.

## Abbreviations

OR: odds ratio
RCT: randomized controlled trial
SDQ: Strengths and Difficulties Questionnaire
SFSW: Strongest Families Smart Website
